# Disparities of *Plasmodium falciparum* infection, malaria-related morbidity and access to malaria prevention and treatment among school-aged children: a national cross-sectional survey in Côte d’Ivoire

**DOI:** 10.1186/1475-2875-14-7

**Published:** 2015-01-05

**Authors:** Clarisse A Houngbedji, Prisca B N’Dri, Eveline Hürlimann, Richard B Yapi, Kigbafori D Silué, Gotianwa Soro, Benjamin G Koudou, Cinthia A Acka, Serge-Brice Assi, Penelope Vounatsou, Eliézer K N’Goran, Agathe Fantodji, Jürg Utzinger, Giovanna Raso

**Affiliations:** Unité de Formation et de Recherche Sciences de la Nature, Université Nangui Abrogoua, 02 BP 801, Abidjan 02, Côte d’Ivoire; Département Environnement et Santé, Centre Suisse de Recherches Scientifiques en Côte d’Ivoire, 01 BP 1303, Abidjan 01, Côte d’Ivoire; Department of Epidemiology and Public Health, Swiss Tropical and Public Health Institute, P.O. Box, CH-4002 Basel, Switzerland; University of Basel, Petersplatz 1, CH-4003 Basel, Switzerland; Unité de Formation et de Recherche Biosciences, Université Félix Houphouët-Boigny, 22 BP 522, Abidjan 22, Côte d’Ivoire; Programme National de Santé Scolaire et Universitaire, 01 BP 1725, Abidjan 01, Côte d’Ivoire; Centre for Neglected Tropical Diseases, Liverpool School of Tropical Medicine, Liverpool, L3 5QA UK; United Nations Children Funds, BP 1650, Bujumbura, Burundi; Institut Pierre Richet de Bouaké, Institut National de Santé Publique, BP 1500, Bouaké, Côte d’Ivoire; Programme National de Lutte contre le Paludisme, Ministère de la Santé et de la Lutte contre le Sida, BP V 4, Abidjan, Côte d’Ivoire

**Keywords:** Malaria, *Plasmodium falciparum*, School-aged children, Self-reported morbidity, Access to prevention and treatment, Cross-sectional survey, Microscopy, Rapid diagnostic test, Côte d’Ivoire

## Abstract

**Background:**

There is limited knowledge on the malaria burden of school-aged children in Côte d’Ivoire. The aim of this study was to assess *Plasmodium falciparum* infection, malaria-related morbidity, use of preventive measures and treatment against malaria, and physical access to health structures among school-aged children across Côte d’Ivoire.

**Methods:**

A national, cross-sectional study was designed, consisting of clinical and parasitological examinations and interviews with schoolchildren. More than 5,000 children from 93 schools in Côte d’Ivoire were interviewed to determine household socioeconomic status, self-reported morbidity and means of malaria prevention and treatment. Finger-prick blood samples were collected and *Plasmodium* infection and parasitaemia determined using Giemsa-stained blood films and a rapid diagnostic test (RDT). Haemoglobin levels and body temperature were measured. Children were classified into wealth quintiles using household assets and principal components analysis (PCA). The concentration index was employed to determine significant trends of health variables according to wealth quintiles. Logistic and binomial negative regression analyses were done to investigate for associations between *P. falciparum* prevalence and parasitaemia and any health-related variable.

**Results:**

The prevalence of *P. falciparum* was 73.9% according to combined microscopy and RDT results with a geometric mean of parasitaemia among infected children of 499 parasites/μl of blood. Infection with *P. falciparum* was significantly associated with sex, socioeconomic status and study setting, while parasitaemia was associated with age. The rate of bed net use was low compared to the rate of bed net ownership. Preventive measures (bed net ownership, insecticide spray and the reported use of malaria treatment) were more frequently mentioned by children from wealthier households who were at lower risk of *P. falciparum* infection. Self-reported morbidity (headache) and clinical morbidity (anaemia) were more often reported by children from less wealthy households.

**Conclusion:**

Seven out of ten school-aged children in Côte d’Ivoire are infected with *P. falciparum* and malaria-related morbidity is considerable. Furthermore, this study points out that bed net usage is quite low and there are important inequalities in preventive measures and treatment. These results can guide equity-oriented malaria control strategies in Côte d’Ivoire.

**Electronic supplementary material:**

The online version of this article (doi:10.1186/1475-2875-14-7) contains supplementary material, which is available to authorized users.

## Background

*Plasmodium falciparum* malaria remains a key global driver of mortality and morbidity with people in sub-Saharan Africa affected most [1, 2]. In Côte d’Ivoire, malaria is the primary cause of consultation in school health services and might be responsible for up to 40% of school absenteeism [3]. According to the world malaria report, the entire population of Côte d’Ivoire is at risk of malaria [2] and *Anopheles gambiae* is the primary vector species [4, 5]. However, there is a strong heterogeneity as wealthier people and those living in urban areas are at lower risk of malaria than poorer counterparts in rural settings [6].

Besides its direct impact on health, malaria places a heavy economic and social burden on endemic countries [7, 8]. Key tools and strategies to fight against malaria include, among others, early diagnosis and treatment with artemisinin-based combination therapy (ACT) and distribution of long-lasting insecticidal nets (LLINs) to populations at risk. While great progress has been registered in the control of malaria in many countries, the burden remains intolerably high in other countries [9]. In Côte d’Ivoire, control efforts by the national malaria control programme are facilitated through continued support from the Global Fund to Fight AIDS, Tuberculosis and Malaria. For example, eight million LLINs were distributed in 2011 and further scaling-up of free LLIN distribution (12 million) to the entire population was planned for the last quarter of 2014.

Here, results are presented from the first national, cross-sectional school-based survey pertaining to parasitic diseases in Côte d’Ivoire, placing particular emphasis on *Plasmodium* infections. The study was carried out in late 2011/early 2012 and involved more than 5,000 children aged five to 16 years [10] and thus provides an up-to-date situation of the extent of *Plasmodium* infection in the school-aged population, associated morbidity, preventive and curative measures and physical access to health systems. The information will be useful for the design of equity-oriented malaria control interventions in Côte d’Ivoire.

## Methods

### Ethics statement

The study protocol received clearance from the ethics committees of Basel (EKBB, reference no. 30/11) and Côte d’Ivoire (reference no. 09-2011/MSHP/CNER-P). Additionally, permission to carry out the study was obtained from the Ministry of National Education. Parents or legal guardians of children provided written informed consent, while children assented orally. All febrile children (tympanic temperature ≥38°C) with a positive rapid diagnostic test (RDT) result for malaria were treated with an ACT, according to World Health Organization (WHO) recommendations and national policies [11]. Anaemic children with a haemoglobin (Hb) level <100 g/l and a negative RDT result were given iron supplementation.

### Study area and sampling procedure

The study was carried out between November 2011 and February 2012 during the dry season and covered the entire Côte d’Ivoire. The country has been stratified into three ecozones [10]. In brief, ecozone 1 in the south is characterised by forest-like vegetation and abundant rainfall; ecozone 2 in the north-eastern part contains savannah-like vegetation and has lower precipitation than the south; and ecozone 3 in the north-western part of the country is relatively small, characterised by savannah-like vegetation, intermediate rainfall and hilly terrain reaching altitudes up to 1,300 m above mean sea level [12].

The study was designed following a lattice plus close pairs sampling approach, as described by Diggle and Ribeiro [13]. The aim was to select approximately 100 locations across Côte d’Ivoire. To apply this design, a lattice indicating latitude and longitude at a unit of 0.5° was overlaid on a map of the country showing the two major ecozones; tropical rainforest in the south (ecozone 1) and the savannah in the north (ecozone 2) [12]. Based on average population densities in the two ecozones, 58 survey locations in the southern ecozone and 42 in the northern ecozone were sampled. While most of these locations were chosen on a regular spacing using the lattice, some locations were chosen at random within a radius of 5 and 20 km from the centre of a lattice location. Due to financial and human resources constraints and in view of recommendations put forward by WHO to sample a minimum of 50 children in surveys aimed at baseline data collection on helminth infection prevalence and intensity, the sample size was restricted to 60 children per school [14]. The inclusion of a sample location was based upon the presence of a school with a minimum of 60 children attending grades 3 to 5. Overall, 94 schools were selected.

### Field and laboratory procedure

The survey team visited one school per day and proceeded as follows. First, the purpose and procedures were explained to school directors and other village authorities. Second, children who had written informed consent from parents/guardians were invited to participate in a parasitological examination and a questionnaire survey. Third, school geographical coordinates were recorded with a hand-held global positioning system (GPS) receiver (Garmin Sery GPS MAP 62; Olathe, USA).

For parasitological assessment of children’s *Plasmodium* infection status, two drops of blood were collected by finger-prick, placed on a microscope slide, and thick and thin blood films prepared. Microscope slides were air-dried, transferred to nearby laboratories where they were stained with Giemsa and examined under a microscope by experienced laboratory technicians for *Plasmodium* species and parasitaemia. The number of parasitised blood cells was counted against 200 leukocytes, assuming a standard count of 8,000 leukocytes per 1 μl of blood. A random sample of 10% of the slides was re-examined by senior laboratory technicians for quality control. In case of discrepancies (e.g. negative *versus* positive results or number of parasites differing by more than 10%), a third technician re-examined the slides and results were discussed until agreement was reached. If the level of discrepancy was less than 10%, the first reading was considered as acceptable. Otherwise, all the slides were re-read. A third drop of blood was subjected to an RDT (ICT ML01 malaria Pf kit; ICT Diagnostics, Cape Town, South Africa). The result of the RDT was read after 15 min according to the manufacturer’s instructions. Finally, a fourth drop of blood was employed to determine Hb levels on a portable HemoCue Hb 301 device (HemoCue AB; Ängelholm, Sweden). Anaemia was determined based on Hb levels, according to WHO recommendations [15]. Anaemia was defined as Hb <115 g/l and Hb <120 g/l for children aged 5–11 years and 12–16 years, respectively. Measurement of body temperature was done using an ear thermometer (Braun ThermoScan IRT 4520; Kronberg, Germany).

A pretested questionnaire was administered to all children to determine household socioeconomic status, self-reported morbidity, self-reported malaria (malaria episode in the last two weeks before the survey), self-reported use of preventive measures and treatment of malaria. This questionnaire had been utilised in a previous school-based survey in Côte d’Ivoire [16]. The questionnaire included a list of 24 household assets (e.g. bicycle, refrigerator and radio), a list of 11 symptoms (e.g. abdominal pain, headache and vomiting) and a list of eight diseases (e.g. malaria, schistosomiasis and skin disease). Children were asked to report any of these symptoms or diseases with a recall period of two weeks. Questions pertaining to preventive measures included the use of bed nets (bed net ownership, children sleeping under a net and children sleeping under a net the night before the survey) and other preventive measures (i.e. use of insecticide spray and other measures that the population considers to prevent the nuisance of mosquitoes or malaria, including fumigating coils, and burning leaves). A question was added to investigate the use of malaria treatment in the previous two weeks.

### Statistical analysis

Data were double-entered and cross-checked using EpiInfo version 3.5.3 (Centers for Disease Control and Prevention, Atlanta, USA). Statistical analyses were done in STATA version 10 (Stata Corporation, College Station, USA). Maps were produced using ArcView GIS version 10.0 (Environmental Systems Research Institute Inc, Redlands, USA).

A higher detectability of the effect of the various variables was observed from categorisation, and hence these variables were categorised for subsequent analyses. Age was categorised into two groups: i) five to ten years, and ii) 11 to 16 years [17]. The distance from school to the nearest health facility was grouped as follows: i) <1 km; ii) 1–5 km; and, iii) >5 km [16]. Schools that were located in villages or towns with a health facility were attributed to the first category. Information on the proximity of sampling schools to the nearest health facility was obtained by the Programme National de Santé Scolaire et Universitaire (PNSSU). Five classes of *Plasmodium falciparum* parasitaemia were considered: i) <50; ii) 50–499; iii) 500–4,999; iv) 5,000-49,999; and, v) >50,000 parasites/μl of blood [18]. For analyses, the combined results of RDT and microscopy were used to determine *P. falciparum* prevalence; an individual was considered as positive for *P. falciparum* if either microscopy or RDT or both tests showed positive results. Microscopy results were employed to assess *P. falciparum* parasitaemia. Logistic and negative binomial regressions were used on *P. falciparum* prevalence and parasitaemia, respectively, to assess associations with different explanatory variables, including sex, age group, socioeconomic status and study setting. A multivariate regression model, adjusted for sex, age group, socioeconomic status and setting, was used to determine how self-reported morbidity, self-reported malaria, clinical morbidity (fever and anaemia), malaria preventive measures and distance from school to the nearest health facility were linked to *P. falciparum* prevalence and parasitaemia. In a further step, random effects at the unit of the school were introduced in the multivariate regression models.

A household asset-based approach was employed to infer socioeconomic status [19]. To weight household assets, principal components analysis (PCA) was used. Household assets were excluded from the list until the first principal component explained more than 30% of the variability. The individuals’ asset scores were summed and ranked according to the total score. Then, the individuals’ total scores were divided into five socioeconomic groups ranging from less wealthy (1) to wealthiest (5) [16, 20]. The concentration index (C-index) was used to evaluate the direction in which self-reported morbidity, clinical morbidity and access to preventive measures and treatment against malaria were associated with socioeconomic groups [21]. A positive C-index is in favour of wealthier households, whereas a negative C-index is in favour of less wealthy households. The *t*-test was used to investigate for statistically significant C-indexes.

## Results

### Compliance and characteristics of study participants

**Figure 1:**
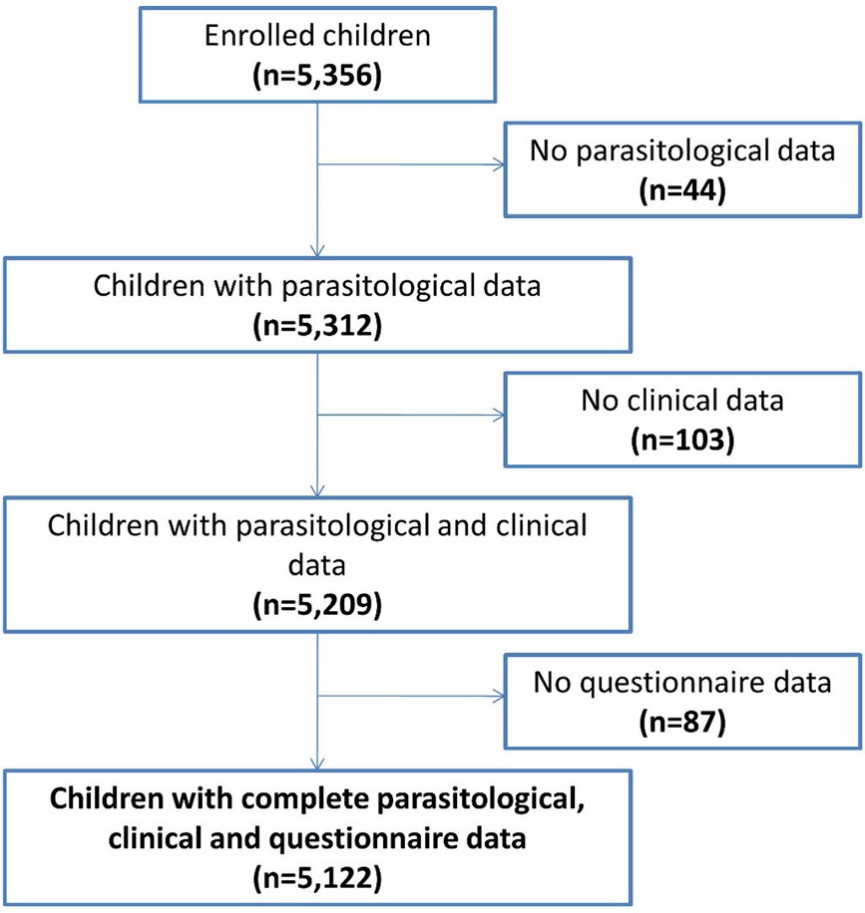
**Flow chart detailing study compliance from a national malaria survey carried out in 93 schools in Côte d’Ivoire between November 2011 and February 2012.**

The study aimed at 60 children in each of the 94 schools selected through a lattice plus close pairs sampling approach across Côte d’Ivoire. One school refused to participate. Overall, 5,356 children were invited to participate in the study. Complete parasitological, clinical and questionnaire data were obtained from 5,122 children (96%). All analyses were done on this cohort (Figure 1). There were significantly more boys than girls (2,714 *versus* 2,408; p <0.001). With regard to age, there were 3,486 children aged five to ten years, while the remaining 1,636 children were aged 11–16 years.

### *Plasmodium falciparum*prevalence and parasitaemia

**Figure 2:**
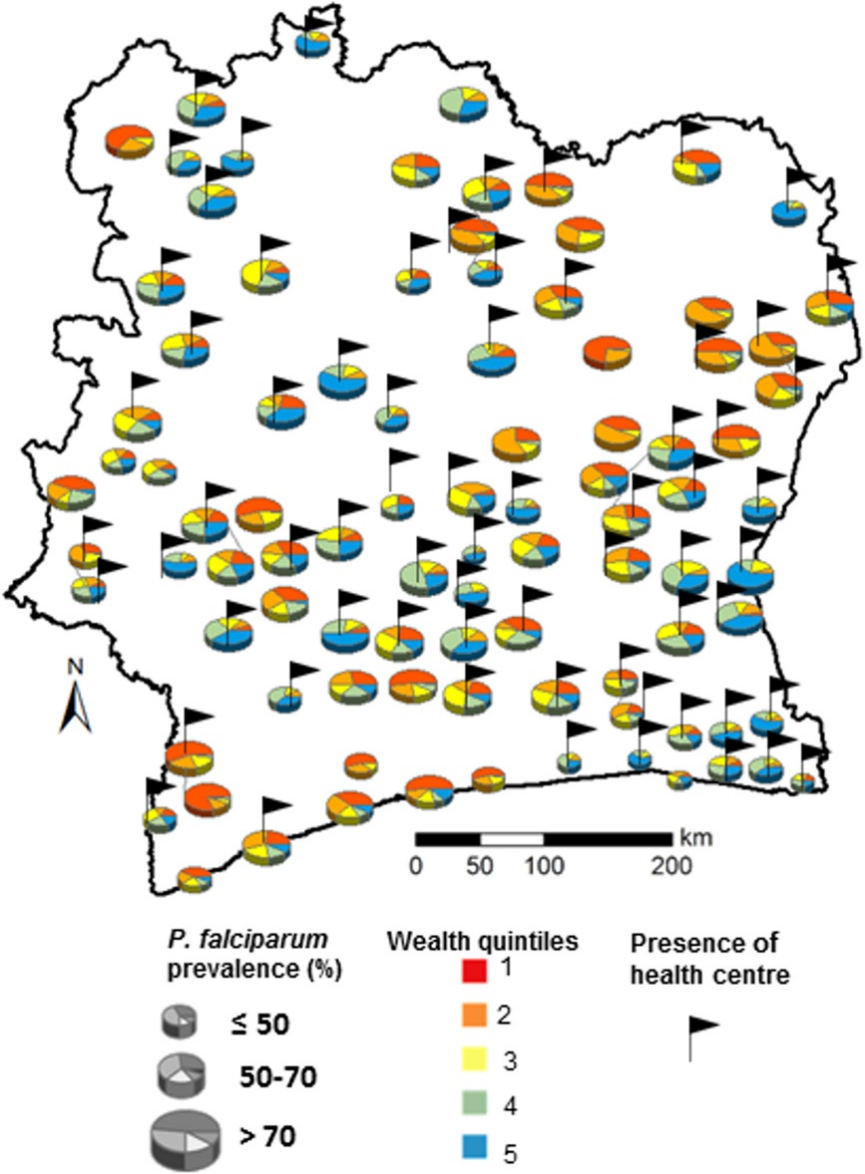
**Survey location map showing children’s household socioeconomic status and** ***Plasmodium falciparum*** **infection prevalence at the unit of the school.** Of note, villages with health facilities are highlighted.

Microscopic examination of thick and thin blood films revealed that 94.5% of the detected *Plasmodium* infections were due to *P. falciparum*, whilst *Plasmodium malariae* and *Plasmodium ovale* accounted for 5.1 and 0.4%, respectively. Further analyses were restricted to *P. falciparum* infection. According to microscopy, 3,539 children were diagnosed with *P. falciparum*, resulting in a prevalence of 69.1%. RDT results revealed slightly fewer children infected (n = 3,425; prevalence = 66.9%), but there was good agreement between the two tests (Kappa = 0.73, standard error (SE) = 0.01). The pooled results from microscopy and RDT found 3,785 *P. falciparum*-infected children, hence an overall prevalence of 73.9%. Figure 2 shows the spatial distribution of *P. falciparum* infection according to the pooled results.

Table 1 shows *P. falciparum* prevalence data according to the pooled microscopy and RDT results, stratified by sex, age group, socioeconomic status and setting. Bivariate logistic regression models with *P. falciparum* results used as outcome variable revealed that boys were significantly more likely to be infected than girls. Furthermore, children from wealthier households were less likely to be infected, as well as children visiting schools in urban settings. Children aged 11–16 years showed a slightly higher *P. falciparum* prevalence compared to their younger counterparts, but the difference lacked statistical significance.

**Table 1:** **Results from bivariate logistic regression models on** ***Plasmodium falciparum*** **prevalence data arising from pooled results with microscopy and RDT**

		Microscopy and RDT		
Variable	Total	No positive (%)	OR (95% CI)	p-value
**Age group (years)**				
5-10	3,486	2,558 (73.4)	1.00	
11-16	1,636	1,227 (75.0)	1.09 (0.95, 1.25)	0.218
**Sex**				
Male	2,714	2,068 (76.2)	1.00	
Female	2,408	1,717 (71.3)	0.78 (0.69, 0.88)	<0.001*
**Socioeconomic status**				
1	1,076	897 (83.4)	1.00	
2	1,011	805 (79.6)	0.78 (0.62, 0.97)	0.028*
3	946	710 (75.1)	0.60 (0.48, 0.75)	<0.001*
4	966	648 (67.1)	0.41 (0.33, 0.50)	<0.001*
5	1,123	725 (64.6)	0.36 (0.30, 0.45)	<0.001*
**Setting**				
Rural	3,958	3,064 (77.4)	1.00	
Urban	1,164	721 (61.9)	0.47 (0.41, 0.55)	<0.001*

Utilised model covariates were age, sex, socioeconomic status and setting.

*Statistically significant (p <0.05).

OR: odds ratio.

CI: confidence interval.

Socioeconomic status: from less wealthy (1) to wealthiest (5).

The geometric mean parasitaemia among infected children was 499 parasites/μl of blood (95% CI: 476–524 parasites/μl of blood) and more than 60% of participants had parasitaemia <500 parasites/μl of blood. Only age was significantly associated with parasitaemia and children belonging to the older age group had significantly lower parasitaemia than their younger counterparts (Table 2).

**Table 2:** ***Plasmodium falciparum*** **parasitaemia stratified by age, sex, socioeconomic status and setting, mean parasitaemia and results from bivariate negative binomial regression models on** ***Plasmodium falciparum*** **parasitaemia**

Variable	Total examined	Positive for ***P. falciparum***infection	***P. falciparum***parasitaemia categories (parasites/μl of blood)						Negative binomial regression model for ***P. falciparum***parasitaemia (parasites/μl of blood)	
			Geometric mean parasitaemia	<50	50-499	500-4,999	5,000-49,999	≥50,000		
				n (%)	n (%)	n (%)	n (%)	n (%)	IRR (95% CI)	p-value
**Age group (years)**										
5-10	3,486	2,397	541.8	1,212 (34.8)	979 (28.1)	1,141 (32.7)	151 (4.3)	3 (0.1)	1.00	
11-16	1,636	1,142	421.0	564 (34.5)	539 (33.0)	475 (29.0)	58 (3.6)	0 (0.0)	0.78 (0.67, 0.90)	0.001*
**Sex**										
Male	2,714	1,942	511.3	875 (32.2)	814 (30.0)	915 (33.7)	109 (4.0)	1 (0.0)	1.00	
Female	2,408	1,597	485.4	901 (37.4)	704 (29.2)	701 (29.1)	100 (4.2)	2 (0.1)	1 (0.87, 1.14)	0.950
**Socioeconomic status**										
1	1,076	855	520.1	268 (24.9)	357 (33.2)	405 (37.6)	45 (4.2)	1 (0.1)	1.00	
2	1,011	755	466.3	294 (29.1)	342 (33.8)	335 (33.1)	40 (4.0)	0 (0.0)	0.90 (0.73, 1.11)	0.314
3	946	664	515.2	312 (33.0)	283 (29.9)	313 (33.1)	37 (3.9)	1 (0.1)	0.95 (0.77, 1.18)	0.642
4	966	595	524.8	402 (41.6)	244 (25.3)	278 (28.8)	42 (4.4)	0 (0.0)	0.81 (0.66, 1.01)	0.061
5	1,123	670	475.4	500 (44.5)	292 (26.0)	285 (25.4)	45 (4.0)	1 (0.1)	0.89 (0.72, 1.09)	0.265
**Setting**										
Rural	3,958	2,870	495.7	1,239 (31.3)	1241 (31.4)	1319 (33.3)	157 (4.0)	2 (0.1)	1.00	
Urban	1,164	669	515.6	537 (46.1)	277 (23.8)	297 (25.5)	52 (4.5)	1 (0.1)	1.02 (0.87, 1.20)	0.800

*Statistically significant (p <0.05).

IRR: incidence rate ratio.

Socioeconomic status: from less wealthy (1) to wealthiest (5).

### Disparities in prevention and treatment against malaria, self-reported morbidity and distance to nearest health facilities across socioeconomic groups

Three-quarter of the children reported to have a bed net at home with children from wealthier households more likely to possess a net. About half of the children reported to sleep regularly under a net and 43% responded that they had slept under a net the night before the survey. Other preventive measures were most frequently reported by children from wealthier households, except for burning leaves. The use of malaria treatment within the past two weeks was more frequently reported by children from wealthier households. With regard to self-reported morbidity, children from poorer households reported significantly more often to have suffered from headache and abdominal pain. Children from poorer household were significantly more diagnosed with anaemia. Detailed results of the relationships and directions between the use of preventive measures, malaria treatment, distance to nearest health facility, self-reported morbidity and schoolchildren’s socioeconomic status are presented in Additional file 1.

### Associations of *Plasmodium falciparum*infection status and parasitaemia with self-reported morbidity

Results from the multivariate regression models used to assess for associations between *P. falciparum* infection status and parasitaemia and self-reported and clinical morbidity, are presented in Table 3. It was found that self-reported vomiting and anaemia were significantly and positively associated with *P. falciparum* infection status. However, children reporting malaria were less likely to be infected with *P. falciparum* than those not reporting malaria. This result remained the same in the random effect logistic regression model after accounting for school location.

**Table 3:** **Results from multivariate regression models on** ***Plasmodium falciparum*** **infection status and parasitaemia with covariates self-reported and clinical morbidity**

	***P. falciparum***infection status				***P. falciparum***parasitaemia (parasites/μl of blood)			
	Logistic regression model		Logistic regression model with random effect		Negative binomial regression model		Negative binomial regression model with random effect	
	Adjusted OR (95% CI)	p-value	Adjusted OR (95% CI)	p-value	Adjusted IRR (95% CI)	p-value	Adjusted IRR (95% CI)	p-value
**Self, reported morbidity/disease**								
Headache	1.15 (0.99, 1.33)	0.077	1.09 (0.93, 1.28)	0.300	1.13 (0.97, 1.33)	0.124	1.06 (0.98, 1.14)	0.136
Hot body	1.00 (0.86, 1.18)	0.953	0.96 (0.81, 1.32)	0.622	1.36 (1.15, 1.61)	<0.001*	1.01 (0.94, 1.10)	0.758
Abdominal pain	1.14 (0.99, 1.32)	0.078	1.10 (0.95, 1.28)	0.216	0.93 (0.80, 1.09)	0.401	1.07 (1.00, 1.16)	0.053
Vomiting	1.25 (1.07, 1.45)	0.004*	1.30 (1.11, 1.52)	0.001*	1.24 (1.05, 1.45)	0.010*	1.16 (1.08, 1.25)	<0.001*
Fatigue	1.00 (0.87, 1.16)	0.952	1.01 (0.87, 1.18)	0.854	0.90 (0.78, 1.05)	0.185	1.01 (0.94, 1.08)	0.855
Loss of appetite	0.96 (0.83, 1.12)	0.608	0.95 (0.81, 1.12)	0.540	0.85 (0.73, 1.00)	0.046*	0.96 (0.89, 1.03)	0.245
Malaria	0.82 (0.70, 0.95)	0.011*	0.80 (0.67, 0.94)	0.008*	1.00 (0.85, 1.18)	0.984	0.89 (0.82, 0.96)	0.004*
**Clinical morbidity**								
Fever	1.04 (0.61, 1.79)	0.881	1.03 (0.58, 1.82)	0.928	3.31 (1.88, 5.81)	<0.001*	1.35 (1.05, 1.73)	0.020*
Anaemia	1.55 (1.34, 1.80)	<0.001*	1.64 (1.40, 1.93)	<0.001*	1.36 (1.17, 1.58)	<0.001*	1.24 (1.16, 1.33)	<0.001*

*Statistically significant (p <0.05).

OR: odds ratio.

IRR: incidence rate ratio.

Models were adjusted for age, sex, socioeconomic status and setting.

With regard to parasitaemia, self-reported hot body and vomiting were positively, and loss of appetite negatively associated with *P. falciparum* parasitaemia. Clinical morbidities (i.e. fever and anaemia) were positively associated with *P. falciparum* parasitaemia. The fever incidence rate was 3.3 higher with increasing parasitaemia levels. After accounting for school location in the multivariate negative binomial model, self-reported vomiting, fever and anaemia still showed significant positive associations with *P. falciparum* parasitaemia. Additionally, self-reported malaria showed a significant negative association to parasitaemia.

### Associations of *Plasmodium falciparum*infection status and parasitaemia with preventive measures against malaria and distance to nearest health facility

Results from the multivariate regression models to determine associations between *P. falciparum* infection status and parasitaemia with preventive measures against malaria and distance to nearest health facility are shown in Table 4. While the use of insecticide spray was negatively associated with *P. falciparum* infection, burning leaves was associated with higher odds of *P. falciparum* infection. Furthermore, attending schools at distances >5 km from the nearest health facility was negatively associated with *P. falciparum* infection. The same findings were observed in both multivariate logistic regressions models (before and after accounting for random effects at the unit of the school) for insecticide spray and burning leaves.

**Table 4:** **Results from multivariate regression models on** ***Plasmodium falciparum*** **infection status and parasitaemia with covariates on prevention and treatment against malaria, and distance to nearest health facility**

	***P. falciparum***infection status				***P. falciparum***parasitaemia (parasites/μl of blood)			
	Logistic regression model		Logistic regression model with random effect		Negative binomial regression model		Negative binomial regression model with random effect	
	Adjusted OR (95% CI)	p-value	Adjusted OR (95% CI)	p-value	Adjusted IRR (95% CI)	p-value	Adjusted IRR (95% CI)	p-value
**Net usage**								
Bed net ownership	1.01 (0.85, 1.20)	0.911	0.97 (0.81, 1.16)	0.747	0.88 (0.73, 1.05)	0.155	1.00 (0.92, 1.09)	0.958
Children sleeping under a net	0.99 (0.76, 1.29)	0.950	1.03 (0.78, 1.37)	0.814	1.09 (0.83, 1.44)	0.545	1.04 (0.91, 1.19)	0.529
Children slept under a net last night	0.99 (0.76, 1.28)	0.936	0.90 (0.69, 1.18)	0.449	0.98 (0.74, 1.29)	0.881	0.95 (0.83, 1.08)	0.447
**Other preventive measures against malaria**								
Fumigating coil	1.14 (0.99, 1.32)	0.074	1.20 (1.03, 1.40)	0.018*	1.06 (0.91, 1.24)	0.459	1.09 (1.01, 1.17)	0.029*
Insecticide spray	0.78 (0.68, 0.90)	0.001*	0.83 (0.71, 0.97)	0.017*	0.95 (0.81, 1.12)	0.570	0.84 (0.78, 0.91)	<0.001*
Smoke by burning leaves	1.26 (1.07, 1.49)	0.006*	1.26 (1.05, 1.51)	0.013*	1.06 (0.90, 1.26)	0.478	1.06 (0.98, 1.15)	0.168
**Malaria treatment**	0.85 (0.72, 1.00)	0.051	0.83 (0.70, 0.99)	0.036*	0.97 (0.81, 1.17)	0.775	0.93 (0.85, 1.02)	0.112
**Distance to nearest health facility** ^**a**^								
<1 km	1.00		1.00		1.00		1.00	
1-5 km	0.76 (0.61, 0.94)	0.012*	0.80 (0.49, 1.29)	0.357	0.71 (0.57, 0.90)	0.005*	0.96 (0.85, 1.08)	0.451
>5 km	0.77 (0.64, 0.92)	0.005*	0.78 (0.52, 1.16)	0.219	0.80 (0.66, 0.96)	0.019*	0.86 (0.78, 0.94)	0.002*

*Statistically significant (p < 0.05).

^**a**^Distance to nearest health facility (<1 km as reference).

OR: odds ratio.

IRR: incidence rate ratio.

Models were adjusted for age, sex, socioeconomic status and setting.

Random effects were introduced for school location.

Only the use of insecticide spray was associated with a significantly lower level of parasitaemia after accounting for school location in the multivariate negative binomial regression model. Children visiting schools located farther away from health facilities had, on average, lower levels of parasitaemia than their counterparts living in close proximity.

## Discussion

This is the first national, cross-sectional, school-based survey conducted in Côte d’Ivoire that investigated *Plasmodium* infection patterns, malaria morbidities and people’s preventive and curative measures. The study was carried out over a four-month period in the dry season towards the end of 2011 and early 2012. This timing allowed the minimisation of logistic and operational challenges, such as high rate of school absenteeism and inaccessibility to the most isolated localities during the rainy season. *Plasmodium falciparum* was the predominant species with a very high overall prevalence (73.9%), despite efforts in place by the national malaria control programme. A decade of socio-political crisis, which further deteriorated an already weak health system, is likely to have played a major role in this finding [22].

In the current study, microscopy revealed a slightly higher prevalence than RDT (69.1 *versus* 66.9%) suggesting a small proportion of false negative results revealed by RDT. This result is somewhat surprising, as RDT is a device that detects malaria parasite antigen in a small amount of blood with monoclonal antibodies impregnated on a test strip [23]. The RDT used in the present study is based on histidine-rich protein 2 (HRP-2). Although this antigen persists in the patient’s blood for weeks after successful antimalarial treatment, it has been suggested to be more sensitive in detecting low-level, fluctuating parasitaemia in chronic malaria [24]. The sensitivity of the RDT employed here is above 95%; however, prior studies have shown that RDT sensitivity declines at parasitaemia levels below 500 parasites/μl of blood to 83% [25]. In this study, over 60% of participants had parasitaemia <500 parasites/μl of blood, which might explain the slightly lower sensitivity of the RDT compared to microscopy. It should, however, be noted that a good agreement was found between microscopy and RDT.

Significant differences were found in *P. falciparum* prevalence between males and females, socioeconomic groups (less wealthy *versus* wealthier households) and place of residence (rural *versus* urban settings). Hence, findings reported here are in line with previous observations made elsewhere in Africa [17, 26–29] and confirm that malaria is a poverty-related disease [7] and that urbanisation is negatively associated with malaria transmission, morbidity and mortality [30]. No significant association was found between age and *P. falciparum* infection status, which is in contrast to other studies [28]. However, children aged 11–16 years showed significantly lower levels of parasitaemia than their younger counterparts. In high endemicity areas, the early exposition to mosquito bites builds up a partial immunity, which in turn results in lower levels of *P. falciparum* infection and parasitaemia with age [31].

With regard to prevention and treatment against malaria, results reported here confirm previous investigations in Côte d’Ivoire and elsewhere, as the use of preventive measures and availability of anti-malarial drugs was associated with the ability to afford the related costs [32–34]. The low bed net use among schoolchildren is consistent with previous findings that people avoid to sleep under a net because of perceived discomfort as highlighted in a recent study from central Côte d’Ivoire [34]. It may also be explained by systematic non-compliance by certain household members [35]. In Côte d’Ivoire, such patterns are likely to occur given past and contemporary malaria control strategies that primarily targeted children below the age of five years and women of childbearing age. These results thus confirm the need for scaling up interventions to other population groups, including school-aged children [34]. The National Malaria Control Programme in Côte d’Ivoire currently places high priority on the distribution of LLINs to cover the entire at-risk population, facilitated by additional funds from the Global Fund to Fight AIDS, Tuberculosis and Malaria. These findings emphasise an existing gap between net ownership and usage, and thus call for additional studies to deepen the current understanding of LLINs among the population, so that control programmes can further improve community effectiveness using bed nets as a major malaria control strategy [36].

Regarding self-reported and clinical morbidity, children from less wealthy households reported significantly more often morbidities such as headache and abdominal pain, and were more frequently diagnosed with anaemia. However, the significant difference in self-reported headache and abdominal pain could be due to the large sample size of the study and might be clinically less relevant. As in previous studies [37, 38], positive associations between *P. falciparum* infection and parasitaemia with self-reported vomiting was found. However, it is important to highlight that all reported morbidities observed in the current study would not necessarily result from *P. falciparum* infections since the self-reported data used were among all children and not only among children with *P. falciparum* infection. Furthermore, those who reported to have suffered from malaria two weeks before the current survey were less likely to have a *P. falciparum* infection or high parasitaemia suggesting that children likely followed efficacious antimalarial treatment [39, 40]. Related to clinical morbidity, children with anaemia or fever were at higher odds of *P. falciparum* infection or high levels of parasitaemia than non-anaemic or non-feverish children. These findings are again consistent with previous studies that established a link between malaria parasite density and fever, and hence, parasite density might confirm a malaria case in the face of fever [41, 42]. Nonetheless, in patients with a negative test result who present with fever, malaria cannot automatically be ruled out, since parasitaemia can fluctuate and remain undetectable for a certain time [42]. With regard to anaemia, it is well established that the *Plasmodium* erythrocyte stages lead to erythrocyte death, which can result in anaemia [43, 44]. Nonetheless, the aetiology of anaemia is multifactorial and other factors including bioavailability of iron in food and other nutritional deficiencies, other parasitic infections (e.g. hookworm), chronic inflammation and genetic conditions must be considered [45–48].

Another interesting finding is that two out of three children surveyed went to a school where the closest health facility was within a 1-km radius. This means that once at school, most of these children can have access to health care. Surprisingly, children attending schools with health facilities in close proximity were more likely to be infected with *P. falciparum* and had higher levels of parasitaemia than those going to schools where health facilities were further away. The exact reasons for these observations remain to be investigated. It might be speculated that the presence of health facilities in close proximity to schools influences health-seeking behaviour; children who go to schools that are far away from health facilities might stay at home when they are sick, while children living in villages with health facilities might seek care when sick. Geostatistical modelling for prediction of malaria risk within a Bayesian framework [49–51] that looks at which risk factors contribute to the spatial distribution of malaria risk might elucidate key reasons behind this observation.

The study has several limitations. First, the results reported here were obtained during a larger investigation focusing on co-infection patterns of *Plasmodium* and intestinal helminths [10]. Second, considering logistical and financial constraints, the diagnosis of *Plasmodium* infection was based on a single finger-prick blood sample per child. Multiple blood samples might have revealed higher prevalence rates. Nevertheless, a combination of microscopy and RDT was used to enhance diagnostic sensitivity. It would have been interesting to employ additional diagnostic assays, particularly polymerase chain reaction (PCR) methods that are highly sensitive [52, 53] and thus would allow to clarify the false-negative diagnoses obtained with RDT compared to microscopy. Third, the survey was carried out during the dry season when malaria transmission is low [4, 54, 55], which might have resulted in an underestimation of the overall *P. falciparum* prevalence. It is conceivable that the overall *P. falciparum* prevalence among school-aged children is somewhat higher during the rainy season. Fourth, although a recall period of only two weeks was considered in the questionnaire interview, in accordance to previous studies [16], there might still be a recall bias.

## Conclusion

This first national school-based cross-sectional survey confirmed that *P. falciparum* endemicity is high in Côte d’Ivoire. Hence, continued and stronger efforts are still necessary to reduce the intolerable burden of malaria in this West African country. Significant disparities in the prevention and treatment of malaria according to socioeconomic groups are apparent, calling for adapting current control strategies to further enhance equity. Although progress has been registered to increase net coverage in high-risk groups, only a relatively small proportion of children at school age reported to have access to preventive measures, including LLINs, and the actual number of children making regular use of nets is quite low. This calls for concerted efforts to increase access to information and preventive measures in the entire population. Furthermore, improved knowledge on the effect of a list of determinants, including climatic, environmental, socioeconomic and control interventions, on the distribution of *P. falciparum* infection in schools needs to be generated through rigorous monitoring platforms. Finally, geostatistical modelling and prediction of malaria risk as done in previous studies [17, 56] is needed to spatially target control needs.

## Electronic supplementary material

Additional file 1: **Disparities in prevention and treatment against malaria, self-reported and clinical morbidity, and distance to health facilities across socioeconomic groups, as assessed by the concentration index (C-index).** (XLS 36 KB)

## Abbreviations

ACT: Artemisinin-based combination therapy
CI: Confidence interval
GPS: Global positioning system
Hb: Haemoglobin
HRP-2: Histidine-rich protein 2
LLIN: Long-lasting insecticidal net
IRR: Incidence rate ratio
OR: Odds ratio
PCA: Principal components analysis
PCR: Polymerase chain reaction
PNSSU: Programme National de Santé Scolaire et Universitaire
RDT: Rapid diagnostic test
WHO: World Health Organization.
